# Botanical Bioflavonoid Composition from *Scutellaria baicalensis*- and *Acacia catechu*-Protected Mice against D-Galactose-Induced Immunosenescence, and Cyclophosphamide Induced Immune Suppression

**DOI:** 10.3390/nu16183144

**Published:** 2024-09-18

**Authors:** Mesfin Yimam, Teresa Horm, Alexandria O’Neal, Paola Chua, Ping Jiao, Mei Hong, Qi Jia

**Affiliations:** 1 Unigen Inc., 2121 South State Street, Suite #400, Tacoma, WA 98405, USA; pjiao@unigen.net (P.J.); meih@unigen.net (M.H.); qjia@unigen.net (Q.J.); 2 Department of Biology, Pacific Lutheran University, 12180 Park Ave. S, Tacoma, WA 98447, USA; teresamarie522@gmail.com; 3 Seagen Pfizer, 21717 30th Dr SE, Bothell, WA 98021, USA; alexhoneal@gmail.com; 4 Washington State Department of Health, 1610 NE 150th St, Shoreline, WA 98155, USA; chua.paolaa@gmail.com

**Keywords:** immunosenescence, NK cells, T cells, antibody IgA, *Scutellaria baicalensis*, *Acacia catechu*, polyphenols, respiratory protection

## Abstract

Oxidative stress and chronic inflammation create a perpetual cycle in the elderly, where impaired immune function amplifies susceptibility to oxidative damage, and oxidative stress further weakens the immune response. This cycle is particularly detrimental to the respiratory system of the elderly, which is an easy target for constant exogenous harmful attacks during cold/flu season or under heavy air pollution. Herbal medicines that protect respiratory function are seen as safer alternatives to conventional therapies; however, there is limited availability of scientifically validated, safe, and effective natural supplements for these conditions. In this study, we evaluated a standardized bioflavonoid composition, UP446, that contains bioactives from the roots of *Scutellaria baicalensis* and the heartwoods of *Acacia catechu* as a natural and nutritional supplement for its antioxidative and immunoregulatory effects in oxidative stress-accelerated aging and chemically induced immune suppression mouse models. Immunosenescence was induced through the repeated subcutaneous inoculation of D-galactose (D-Gal) at a dose of 500 mg/kg/day in CD-1 mice. UP446 was administered orally at doses of 100 mg/kg and 200 mg/kg starting in the fifth week of immunosenescence induction. This study lasted a total of ten weeks. All mice received a quadrivalent influenza vaccine 2 weeks before termination. Whole blood, serum, spleen homogenate, and thymus tissues were processed for analysis. Cyclophosphamide (Cy)-induced immunosuppression was triggered by three consecutive injections of cyclophosphamide at 80 mg/kg/day, followed by the oral administration of UP446 for 18 days at doses of 100 mg/kg and 200 mg/kg. Blood was collected from each animal at necropsy, and serum was isolated for IgA and IgG ELISA analysis. UP446 was found to improve immune response, as evidenced by the stimulation of innate (NK cells) and adaptive immune responses (T cells and cytotoxic T cells), an increase in antioxidant capacity (glutathione peroxidase), the preservation of vital immune organs (the thymus), and a reduction in NFκB. UP446 also increased serum levels of IgA and IgG. The findings presented in this report demonstrate the antioxidative, anti-inflammatory, and immune-regulatory activities of UP446, suggesting its potential use in respiratory conditions involving immune stress due to aging, oxidative stress, and/or pathogenic challenges.

## 1. Introduction

The pathophysiological association between pollution-induced oxidative stress and lung injury features a complex interplay with significant health implications. Environmental pollutants, such as PM2.5 particulate matter, can induce oxidative stress in the respiratory system [1]. This oxidative stress disrupts the balance between antioxidant defenses and reactive oxygen species (ROS) production, leading to cellular damage and inflammation in the respiratory system, especially the lungs. Oxidative stress is a key driver of inflammatory responses in lung tissues. It activates signaling pathways that promote the release of proinflammatory cytokines, chemokines, and damage-associated molecular patterns (DAMPs) like HMGB1, which collectively exacerbate inflammation and contribute to lung tissue injury [2].

HMGB1, in particular, is highlighted as a critical mediator in this process. It accumulates extracellularly in response to oxidative stress induced by pollutants, further amplifying inflammatory reactions and impairing immune cell functions. The buildup of HMGB1 in the airways and lungs is linked to a greater severity of inflammatory lung injury. The pathophysiology involves a cascade of events where oxidative stress initiates inflammatory responses, leading to tissue damage and compromising lung function. This relationship underscores the need for effective strategies to mitigate pollutant exposure, enhance antioxidant defenses, and potentially target HMGB1 to protect against pollution-induced oxidative stress and mitigate lung injury [3,4].

Previously, Yimam et al. (2023) proposed that reducing extracellular HMGB1 levels could restore immune cell functions and protect the lungs from oxidative stress-induced injuries. They tested their hypothesis using animal models and immune cell cultures exposed to oxidative stress or bacterial challenges [5]. UP446 was found to significantly reduce mortality rates, inhibit proinflammatory cytokines (TNF-α, IL-1β, IL-6) and chemokines (CINC-3), facilitate enhanced bacterial clearance from the lungs, and decrease airway protein levels, notably through the reduction in extracellular HMGB1 levels. Based on these facts, the authors concluded that the botanical composition is a potential intervention to protect the lungs against oxidative stress-induced injuries.

Oxidative stress, arising from an imbalance between antioxidants and reactive oxygen species (ROS), intensifies with age due to cumulative exposure to environmental factors, lifestyle choices, and declining cellular repair mechanisms. As such, the relationship among oxidative stress, old age, and compromised immunity has damaging consequences impacting overall respiratory health and susceptibility to diseases. In particular, the consequences are detrimental for the respiratory system as it is an easy target for constant exogenous harmful attacks during respiration [6].

In an aging population, physiological changes such as decreased antioxidant defenses and mitochondrial dysfunction contribute to heightened oxidative stress. This oxidative burden can impair immune function by disrupting signaling pathways crucial for immune cell activation, proliferation, and response to pathogens. Compromised immunity in aging is characterized by the reduced efficiency of innate and adaptive immune responses. This includes impaired phagocytosis by macrophages, the decreased production of cytokines and antibodies by lymphocytes, the dysregulation of inflammatory processes, and compromised immune responses [7,8]. These alterations make senior adults more vulnerable to infections due to chronic inflammatory conditions, less responsive to vaccination, and recover more slowly from illnesses. Moreover, oxidative stress-induced damage to immune cells and thymus tissues exacerbates age-related immune dysfunction. This can lead to a vicious cycle where impaired immunity further enhances susceptibility to oxidative stress and associated health challenges [9,10]. Therefore, addressing oxidative stress in aging populations through lifestyle interventions, the supplementation of dietary antioxidants, and potentially targeted interventions represents a promising approach to mitigate immune decline and improve overall health outcomes in senior adults.

Dietary supplements containing natural polyphenols offer a distinct advantage in oxidative stress management compared to basic antioxidant vitamins. These polyphenols, characterized by their diverse structures, potentially provide additional benefits beyond simple antioxidation [11]. Extracts of *Scutellaria baicalensis* roots and *Acacia catechu* heartwoods, along with their active bioflavonoid constituents present in the UP446 composition, are recognized for their notably varied antioxidant properties. For instance, baicalin, a flavonoid from the root of *S. baicalensis*, effectively scavenges DPPH free radicals (IC50 = 27.21 µM), inhibits CuSO4-induced lipid peroxidation (IC50 = 95.09 µM), demonstrates significant metal-chelating activity (IC50 = 352.04 µM) [12], and mitigates ROS production and MDA levels, while restoring SOD activity [13]. Similarly, catechins from green teas and the heartwoods of *Acacia catechu* demonstrate potent abilities to neutralize reactive oxygen and nitrogen species, effectively protecting against oxidative stress-induced lipid peroxidation and associated damage. They act as scavengers of reactive oxygen species (ROS) and chelators of metal ions. Additionally, catechins exert indirect antioxidant effects by inducing antioxidant enzymes, inhibiting pro-oxidant enzymes, and stimulating the production of phase II detoxification and antioxidant enzymes [14].

Hence, in the current study, we hypothesized that a standardized bioflavonoid composition, UP446, could mitigate oxidative stress and aging-associated declines in immune response. We proceeded to test this hypothesis using oxidative stress-accelerated immune senescence and chemically induced immune suppression models. Its antioxidation potential was also tested in vitro.

## 2. Materials and Methods

### 2.1. Composition

UP446 is a formulation containing extracts from heartwoods of *Acacia catechu* (*Senegalia catechu*) and roots of *Scutellaria baicalensis*, with preparation details disclosed in a US patent [15]. The process for preparing the extracts and the quantification of their full composition using High-Performance Liquid Chromatography (HPLC) have been described by Yimam et al. [16]. UP446 is a greenish yellow to brown powder with a standardized bioflavonoid composition that contains not less than 60% baicalin from *Scutellaria* as its major component and not less than 10% catechins from *Acacia* plus other minor bioflavonoids from both plants [16].

### 2.2. Animals

Mice (n = 130) (5 per cage) were housed in polypropylene cages and identified individually by tail numbers. Each cage was covered with a wire bar lid and filtered top (Allentown, NJ, USA). Cage cards were used to identify each cage, displaying the project number, test article, dose level, group, and animal numbers. Harlan T7087 soft cob bedding was used and changed at least twice a week. Animals were provided with fresh water and rodent chow diet #T2018 (Harlan Teklad, 370W, Kent, WA, USA) ad libitum, and housed in a temperature-controlled room (21.7–22.2 °C) on a 12 h light–dark shifts. In vivo experiments were carried out in accordance with institutional guidelines, consistent with the Guide for the Care and Use of Laboratory Animals with IACUC #s IAS-AO-030321 and IAS-PC-013123.

### 2.3. Antioxidant Assay by Oxygen Radical Absorbance Capacity (ORAC)

A UP446 sample was diluted in PBS (phosphate-buffered saline) to generate 500, 250, and 50 µg/mL solutions for testing. Two independent dilution series were prepared. A Trolox standard curve was generated to obtain 100, 50, 25, 12.5, and 6.125 µM in PBS. In total, 150 µL of fluorescein dilution was added to each well of a black 96-well plate, and 25 µL of Trolox dilutions or sample dilutions were added to triplicate wells. The plate was incubated at 37 °C for 10 min. AAPH (2,2′-Azodiisobutyramidine dihydrochloride) solution was prepared fresh for the assay using the assay buffer. The assay was initiated by adding 25 µL of the AAPH solution. The plate was inserted into the plate reader and a kinetic analysis of fluorescence decay was monitored every minute for 30 min at 37 °C. Trolox antioxidant activity was used as a standard to compare the test sample dilutions. The area under the curve of normalized decay curves was determined for each concentration of control and sample. The Trolox net AUC (Area Under the Curve) was used as a standard curve to calculate the Trolox equivalence of the test samples. Assay Plates: black 96-well clear bottom plates; reagents: phosphate-buffered saline (Lot: PBS104, ZenBio, Durham, NC, USA); assay buffer fluorescein (Lot: 041019, ZenBio); Trolox (Lot: 010622, ZenBio); AAPH (Lot: 0554100, ZenBio); instruments: Tecan Infinite F500 Plate Reader (Tecan, Männedorf, Switzerland)

### 2.4. Cyclophosphamide-Induced Immunosuppression in Mice

Purpose-bred male CD-1 mice (n = 50) were purchased from Charles River laboratories (Wilmington, MA, USA) at 22–24 g in body weight and acclimated for 1 week. Following acclimation, the animals were weighed for their baseline weights and randomized into 5 groups. There were 10 animals per group. The groups included G1 = control animals without cyclophosphamide, G2 = control model with cyclophosphamide, G3 = cyclophosphamide + levamisole 10 mg/kg, G4 = cyclophosphamide + UP446 100 mg/kg, and G5 = cyclophosphamide + UP446 200 mg/kg. Dosages were adjusted weekly based on bodyweight. Immunosuppression was induced by administering cyclophosphamide (Cy) at 80 mg/kg i.p. for 3 consecutive days. This study lasted for 3 weeks. For days 1–3, cyclophosphamide (Cy) was administered to all groups except the normal control group (−). Cy dissolved in saline was injected intraperitoneally at a 0.2 mL volume per animal. For days 4–21, mice were gavaged orally once per day for the indicated test materials. During the intervention period, the mice received no Cy. Animals were weighed weekly to determine subsequent dosages. Solutions were prepared for treatment groups in 0.5% CMC (Carboxy Methylcellulose) for oral delivery; 0.4 mL was administered per animal orally. On day 22 (Necropsy Day), blood was collected using a serum separator tube and was kept for at least 30 min before spinning at 11,000 rpm for 5 min at 4 °C. The serum was aspirated into microcentrifuge tubes (with about 400 µL yield) and stored in freezer until use.

UP446 was historically evaluated in our lab for multiple indications at 50, 100, 150, 200, and 250 mg/kg. Considering the repeated administration nature of the design, we selected 100 and 200 mg/kg for this study. For cyclophosphamide and levamisole dosages, we referenced previous reports [17]. Per protocol, the mice were monitored for 30 min after each gavage and daily thereafter for behavioral changes, food and water consumption, physical activity, appearance, and discharges from the eyes and orifices.

### 2.5. IgA and IgG ELISA

ELISAs for serum IgA and IgG were carried out following the manufacturer’s instructions. ELISA products Abcam ab157719 and Abcam ab157717 (Abcam, Cambridge, UK) were used for IgG and IgA, respectively. While 0, 12.5, 25, 50, 100, 200, and 400 ng/mL concentrations were used for IgA standards; 0, 3.91, 7.81, 15.6, 31.3, 62.5, 125, and 250 ng/mL concentrations were used for IgG. Briefly, 100 μL of each standard and samples were pipetted in duplicate into predesignated wells. The micro titer plate was incubated at room temperature for sixty (60 ± 2) minutes. In total 100 μL of 1× enzyme–antibody conjugate was washed and added to each well, and incubated at room temperature for thirty (30 ± 2) minutes. The wells were washed and blotted. In total, 100 μL of TMB Substrate Solution was pipetted into each well and incubated in the dark at room temperature for ten minutes. After ten minutes, 100 μL of Stop Solution was added to each well, and the absorbance (450 nm) of the contents of each well was determined.

### 2.6. D-Galactose-Induced Accelerated Immune Senescence Model

Twelve-week-old purpose-bred male CD-1 mice (n = 80) were obtained from Charles River Laboratories, Inc. (Wilmington, MA, USA), and employed for the study post a one-week acclimation period. They were randomly divided into four groups of immunized and non-immunized groups. The groups were as follows: G1 = normal control + vehicle (0.5% CMC), G2 = D-galactose + vehicle, G3 = D-galactose + UP446 100 mg/kg, and G4 = D-galactose + UP446 200 mg/kg. Each group consisted of 10 mice. Dosages were adjusted weekly based on bodyweight.

To induce accelerated immune senescence, mice were subcutaneously injected with D-galactose at a dosage of 500 mg/kg daily for 10 weeks. Treatment began four weeks after the induction started, at the beginning of week 5, with two doses of UP446 (100 mg/kg for the low dose and 200 mg/kg for the high dose), administered orally in a 0.5% CMC suspension for both the immunized and non-immunized groups. In week 8, all mice except those in the non-immunized groups received an intramuscular injection of 3 μg of the Fluarix quadrivalent influenza seasonal vaccine (2020–2021) (Lot # UJ477AA, 2020–2021) from GSK. This vaccine contained 60 μg of hemagglutinin per 0.5 mL single human dose, formulated with 15 μg of each of the four influenza strains: H1N1, H3N2, B-Victoria lineage, and B-Yamagata lineage.

Per the protocol, the mice were monitored for 30 min after each gavage and daily thereafter for behavioral changes, food and water consumption, physical activity, appearance, and discharges from the eyes and orifices.

The daily oral gavaging of UP446 was performed for six weeks, from the fifth week to the tenth week. At the time of necropsy, 14 days after immunization, 1 mL of blood was collected. A 110 μL aliquot was taken for a flow cytometry immunity panel in BD Microtainer^®^ Tubes with Dipotassium EDTA (Lavender; BD, Franklin Lakes, NJ, USA). Serum was separated from the rest of the blood using SST and centrifuged at 1500 rpm for 10 min at 4 °C, yielding approximately 400 μL for antibody ELISAs and enzymatic assays. The thymus weights were recorded for every animal to determine thymus indices. The spleens were preserved on dry ice at necropsy and then transferred to −80 °C for future use.

### 2.7. Flow Cytometry Method

Whole blood was collected via cardiac puncture and transferred to EDTA blood collection tubes. Non-immunized samples were refrigerated overnight, while immunized samples were kept at room temperature on a nutator for up to 6 h. The samples were then transported on ice to the Flow Contract Site Lab (Bothell, WA, USA) for a 10-marker flow cytometry analysis. Doublets and debris were excluded, and the total cell population was identified using forward and side scatter gating. Fluorescent antibodies were used to distinguish cell populations. The blood was incubated for 15–20 min with the following antibodies: Mouse (m)CD45 V510 clone 30-F11 (BioLegend, San Diego, CA, USA, #103137), mCD3 APCCy7 clone 17A2 (BioLegend, #100221), mCD4 PECy7 clone GK1.5 (BioLegend, #100421), mCD8 AF700 clone 53-6.7 (BioLegend, #100729), mCD49b FITC clone DX5 (BioLegend, #108905), mLy6G APC clone 1A8 (BioLegend, #127613), mCD45R/B220 V605 clone RA3-6B2 (BioLegend, #103243), mNKp46 V421 clone 29A1.4 (BioLegend, #137611), mTCRγδ PE clone GL3 (BioLegend, #118107), and 7-AAD (BD or BioLegend, #420403). Red blood cells were lysed using PharmLyse (BD, #555899), and the samples were then centrifuged and resuspended in calcium- and magnesium-free DPBS (ThermoFisher Scientific, Waltham, MA, USA, #J67802K2) before being mixed with CountBright beads (ThermoFisher Scientific, #C36950). Flow cytometric data were acquired using a FACSCantoA, FACSCanto 10 Color flow cytometer (BD, Franklin Lakes, NJ, USA), and BD FACSDiva™ software version 9.0. Cell populations were determined by electronic gating based on the forward versus side scatter.

### 2.8. Serum Glutathione Peroxidase Activity

Mouse serum samples were tested for glutathione peroxidase activity using a Glutathione Peroxidase Assay Kit (Colorimetric) (Abcam ab102530) following the manufacturer’s instructions. Briefly, a reaction mix was prepared by adding 33 µL of Assay Buffer, 3 µL of 40 mM NADPH solution, 2 µL of GR solution, and 2 µL of GSH solution. While standard wells were prepared at 100 µL standard dilutions; sample and reagent control wells were prepared at 50 µL per well. In total, 40 µL of Reaction Mix was added to the sample and reagent control wells only. The plate was incubated at room temperature for 15 min. The absorbance was read at OD 340 nm. In total, 10 µL of cumene hydroperoxide solution was added to the sample and reagent control wells only. The output (A1) was measured on a microplate reader at T1 at OD340 nm, and then incubated at 25 °C for 5 min, protected from light. The output (A2) was measured on a microplate reader at T2 at OD340 nm. Each sample was assayed in duplicate, and the absorbances of each sample were compared to a NADPH Standard curve.

### 2.9. Spleen Homogenate NFκB

Spleen tissues from the mice were homogenized in RIPA Lysis Buffer (50 mM Tris-HCl, pH 7.6, 150 mM NaCl, 1% NP-40, 0.5% Sodium deoxycholate, 0.1% SDS) at a concentration of 100 mg of tissue per mL of buffer, with three 10 s cycles on ice. The homogenates were then incubated on ice for 30 min to facilitate tissue lysis, followed by centrifugation at 16,000 RCF for 10 min at 4 °C. The supernatants were collected into new microcentrifuge tubes and stored at −80 °C. Spleen homogenates were subjected to SDS-PAGE, and the expression of markers was analyzed using primary antibodies against NFκB (Abcam, ab140751) with a Goat anti-Rabbit IgG Alexa Fluor Plus 488 secondary antibody, and anti-β-actin (Life Technologies, Carlsbad, CA, USA, MA5-15739-D680).

### 2.10. Serum Advanced Glycation End Products (AGEs)

Mouse serum samples were tested for AGEs using the Advanced Glycation End Product (AGE) Assay Kit (Colorimetric Assay, Abcam ab273298) according to the manufacturer’s instructions. Briefly, samples were diluted to a protein concentration of 1 mg/mL with AGE Assay Buffer. In total, 10 µL of the diluted samples was added to the wells of a white 96-well plate, and the volume was adjusted to 200 µL with AGE Assay Buffer. A 1 mg/mL AGEs Positive Control was prepared by adding 2 µL of 10 mg/mL AGE Positive Control to 18 µL of AGE Assay Buffer. The same volume of the 1 mg/mL AGE Positive Control was added to the wells designated as “Positive Control”, and the volume was adjusted to 200 µL with AGE Assay Buffer. The plate was incubated at room temperature for 5 min, protected from light. Fluorescence was measured at room temperature in end-point mode using a Tecan fluorescence microplate reader with excitation/emission settings of 360/460 nm.

### 2.11. Statistical Analysis

All descriptive data analyses were carried out using SigmaPlot 14.5 DA software (Palo Alto, CA, USA). The results are presented as the mean ± SD. Statistical significance among groups was assessed using single-factor analysis of variance and Student’s *t*-test. *p*-values of 0.05 or less (*p* ≤ 0.05) were regarded as statistically significant. Percent changes from the vehicle were determined using the following formula: % change = {(Mean value of Vehicle − Mean value of active test article)/Mean value of Vehicle} × 100. Flow cytometry data are reported as a percentage of all live white blood cells (CD45+ cells).

## 3. Results

### 3.1. Antioxidation Activity of UP446 as Measured by ORAC

UP446 has been found to possess strong antioxidation activity as measured in ORAC with a total ORAC value of 70,930 (µmole TE) per gram (Table 1). The ability of UP446 to neutralize Peroxyl Radicals, Hydroxyl Radicals, Peroxy Nitrite, Super Oxide Anion, and Singlet Oxygen radicals was measured and found as described in Table 1.

The ORAC result is expressed as the micromole Trolox equivalency (µmole TE) per gram. The ORAC (Trolox equivalents, TE) value was calculated by dividing the area under the sample curve by the area under the Trolox curve, with both areas being corrected by subtracting the area under the blank curve. Trolox was used as a positive control.

### 3.2. Immunomodulation Effect of UP446 on Cyclophosphamide-Induced Immunosuppression

All mice survived the duration of the study with no apparent changes suggestive of toxicity.

**Figure 1:**
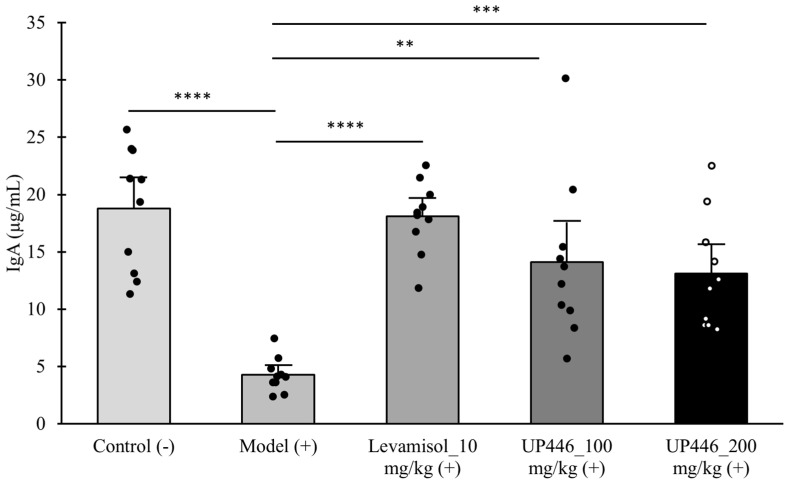
Immunomodulation effect of UP446 on cyclophosphamide-induced immunosuppression. Immunosuppression was induced by administering cyclophosphamide (Cy) at 80 mg/kg intraperitoneal for 3 subsequent days to CD-1 mice. Male CD-1 mice (n = 10) were treated with Levamisol_at 10 mg/kg, and UP446 at 100 and 200 mg/kg starting from day 4 for 18 days. The study lasted for 3 weeks. The control (−) group without cyclophosphamide (Cy) and model (+) received the vehicle 0.5% CMC (Carboxy Methylcellulose). The serum was separated at necropsy, and ELISA was carried out for IgA following the manufacturer’s instructions. Data are expressed as the mean ± SD. ** *p* ≤ 0.01 vs. model (+); *** *p* ≤ 0.001 vs. model (+); **** *p* ≤ 0.0001 vs. model (+). (+) represents model induction.

**Figure 2:**
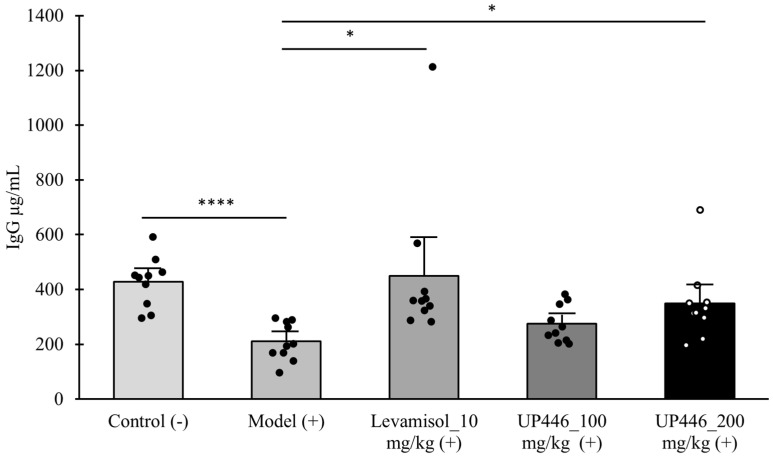
Immunomodulation effect of UP446 on cyclophosphamide-induced immunosuppression. Immunosuppression was induced by administering cyclophosphamide (Cy) at 80 mg/kg intraperitoneal for 3 subsequent days to CD-1 mice. Male CD-1 mice (n = 10) were treated with Levamisol_at 10 mg/kg, and UP446 at 100 and 200 mg/kg starting from day 4 for 18 days. The study lasted for 3 weeks. The control (−) group without Cy and model (+) received the vehicle 0.5% CMC (Carboxy Methylcellulose). The serum was separated at necropsy, and ELISA was carried out for IgG following the manufacturer’s instructions. Data are expressed as the mean ± SD. * *p* ≤ 0.05 vs. model (+); **** *p* ≤ 0.0001 vs. model (+). (+) represents model induction.

The injection of Cy to mice resulted in 4.38- and 2.03-fold significant reductions in the serum levels of IgA and IgG in CD-1 mice, respectively (Figure 1 and Figure 2). These reductions were restored by the oral supplementation of UP446. In the case of IgA, mice administered with UP446 at 100 mg/kg and 200 mg/kg exhibited 3.26- and 3.06-fold increases in the serum IgA levels of mice, respectively (Figure 1). These increases in the level of serum IgA were statistically significant compared to the immune-suppressed group. In comparison to the controls, normal control and levamisole mice treated with the lower dosages (100 mg/kg UP446) showed no statistical significance difference, while the changes for the high dose (200 mg/kg) were significantly lower, *p* = 0.02.

A similar immune modulation was observed for IgG. Animals administered with UP446 at 100 and 200 mg/kg showed 1.3- and 1.66-fold increases in the level of serum IgG, respectively. The induction in IgG was statistically significant for the 200 mg/kg of UP446 (Figure 2). The reference compound (levamisole) produced statistically significant 4.22- and 2.13-fold increases in the level of serum IgA and IgG, respectively. Both the 100 and 200 mg/kg UP446-treated groups showed no significant difference against levamisole; however, in comparison to the normal control, the low-dose group showed a statistically significant lower efficacy with no difference in efficacy to that of the high dose UP446.

### 3.3. Effect of UP446 on the Immune Organ: The Thymus

All mice survived the duration of the study with no apparent changes suggestive of toxicity.

**Figure 3:**
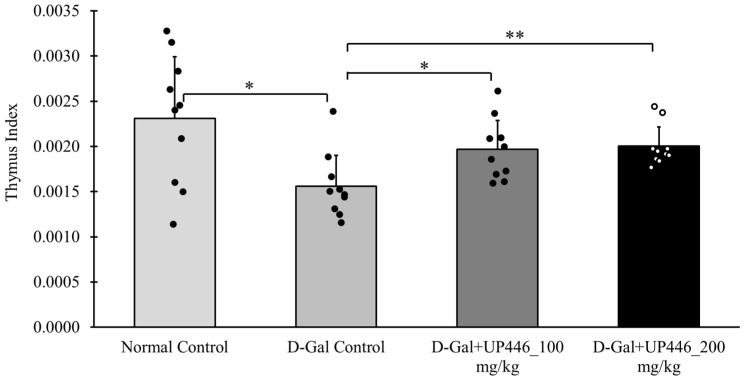
Impact of UP446 on the immune organ in immunized mice. Male CD-1 mice (n = 10) were inoculated subcutaneously with D-galactose at a dose of 500 mg/kg daily for 10 weeks to induce accelerated aging. Starting in week 5, mice were treated orally with UP446 at doses of 100 and 200 mg/kg. They were immunized at the end of week 8 (beginning of week 9), and necropsy was performed at the end of week 10 (14 days post immunization). The normal control group, which did not receive D-galactose, and the D-galactose control group were both administered the vehicle, 0.5% CMC (Carboxy Methylcellulose). Thymus weights were measured for each animal to calculate thymus indices. Data are expressed as the mean ± SD * *p* ≤ 0.05 vs. D-gal control; ** *p* ≤ 0.01 vs. D-gal control. D-Gal: D-galactose.

In the immunized mice, D-Gal-induced immune senescence mice administered the vehicle displayed a significant 30.3% reduction in the thymus index compared to the normal control mice (Figure 3). This reduction was reversed by UP446 at both dosages. Mice treated orally with UP446 at 100 mg/kg and 200 mg/kg showed a 47.4% and 49.4% increase in the thymus index, respectively, compared to the vehicle-treated immune-compromised group (Figure 3). This reversal was statistically significant for both doses of UP446. These observations were comparable to the normal control, as there was no statistical difference between the normal control and UP446-treated mice for both the dosages.

**Figure 4:**
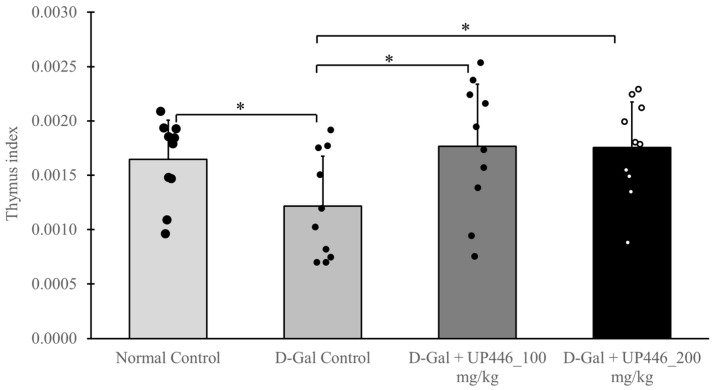
Impact of UP446 on the immune organ in non-immunized mice. Male CD-1 mice (n = 10) were inoculated subcutaneously with D-galactose at a dose of 500 mg/kg daily for 10 weeks to induce rapid aging. Starting in week 5, the mice received oral treatment with UP446 at doses of 100 and 200 mg/kg. Necropsy was performed at the end of week 10. The normal control group, which did not receive D-galactose, and the D-galactose control group were administered the vehicle, 0.5% CMC (Carboxy Methylcellulose). Thymus weights were recorded for each animal to determine thymus indices. Data are expressed as the mean ± SD. * *p* ≤ 0.05 vs. D-gal control. D-Gal: D-galactose.

Likewise, in non-immunized mice, UP446 at doses of 200 mg/kg and 100 mg/kg resulted in statistically significant increases in the thymus index, with increases of 27.4% and 31.6%, respectively, compared to the vehicle-treated immune-compromised mice (Figure 4). These observations were comparable to the normal control, as there was no statistical difference between the normal control and UP446-treated mice for both the dosages.

### 3.4. Impact of UP446 on Circulating Immune Cell Populations in Immunized Mice

The levels of circulating immune cells in the immunized groups of mice were assessed at the end of the study. Conveyed as a percentage of entire WBCs (CD45+ cells), the dosage correlated with 22.5% and 42.5% increases in natural killer cells (NK-cells), as recorded for mice administered UP446 at 100 and 200 mg/kg, respectively. These elevations of circulating NK-cells were statistically significant when compared to the D-gal + vehicle group. Changes for other immune cells were minimal (Table 2).

### 3.5. Impact of UP446 on Circulating Immune Cell Populations in Non-Immunized Mice

Compared to the D-gal non-immunized group, the non-immunized group receiving 200 mg/kg of UP446 + D-gal showed a statistically significant 21.5% increase in T cell levels (CD3+ cells), conveyed as a percentage of total WBCs (CD45+ cells) (Table 3). A similar increase of 37.5% was also observed in CD3+CD8+ cytotoxic T cells in non-immunized D-gal mice treated with 200 mg/kg of UP446 (Table 3) At the lower dosage of 100 mg/kg, non-immunized D-gal mice administered UP446 exhibited a statistically significant 41.2% increase in natural killer cells (Table 3). Statistically non-significant moderate to minimal changes were observed for other immune cells at both dosages.

### 3.6. Impact of UP446 on a Transcription Factor NFκB

**Figure 5:**
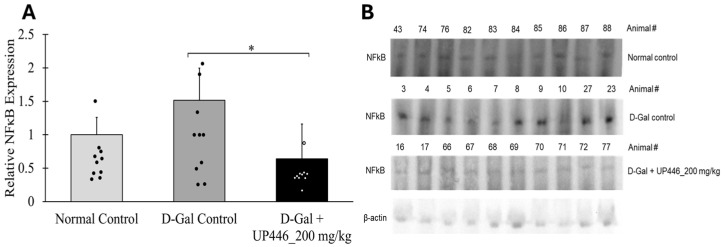
Effect of UP446 on a transcription factor NFκB on non-immunized mice. Male CD-1 mice (n = 10) were inoculated subcutaneously with D-galactose at a dose of 500 mg/kg daily for 10 weeks to induce rapid aging. Starting in week 5, the mice were treated orally with UP446 at a dose of 200 mg/kg. Necropsy was performed at the end of week 10. The normal control group, which did not receive D-galactose, and the D-galactose control group were administered the vehicle, 0.5% CMC (Carboxy Methylcellulose). During necropsy, spleens were placed on dry ice and subsequently stored at −80 °C for future use. Spleen homogenates were subjected to SDS-PAGE, and marker expressions were detected using primary antibodies against NFκB. (**A**) = Data are expressed as the mean ± SD. (**B**) = SDS-PAGE Gel. * *p* ≤ 0.05 vs. D-gal control. D-Gal: D-galactose.

The SDS-PAGE assay showed that non-immunized D-gal mice treated with the vehicle had a 51.6% increase in the expression of NFκB. This elevation in the expression of the inflammatory transcription factor was mitigated by the use of UP446. A statistically significant 57.9% reduction in NFκB expression was observed in the non-immunized mice treated with 200 mg/kg of UP446 (Figure 5). When the reduction was compared to the normal control mice, it was not statistically significant.

### 3.7. Effect of UP446 on Advanced Glycation End Products (AGEs)

**Figure 6:**
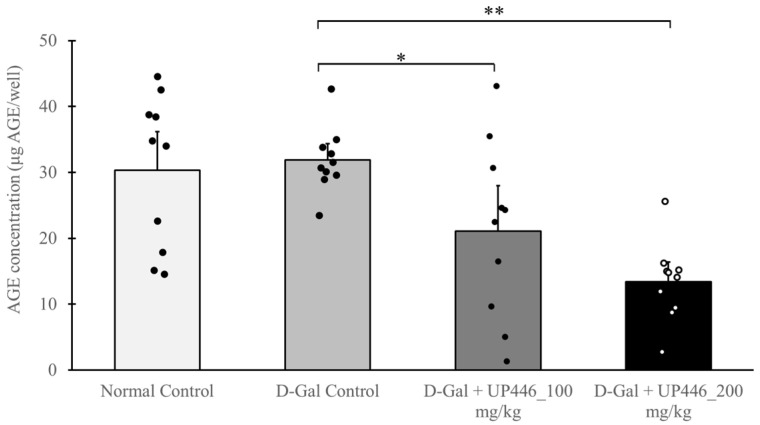
Effect of UP446 on advanced glycation end products (AGEs) on non-immunized mice. Male CD-1 mice (n = 10) received daily subcutaneous inoculation of D-galactose at 500 mg/kg for 10 weeks to induce rapid aging. Beginning in week 5, the mice were treated orally with UP446 at a dose of 200 mg/kg. Necropsy was performed at the end of week 10. The normal control group without D-gal and D-Gal control received the vehicle 0.5% CMC (Carboxy Methylcellulose). The serum was separated at necropsy and tested for AGEs using the Advanced Glycation End Products (AGEs) Assay Kit according to the manufacturer’s instructions. Data are expressed as the mean ± SD. * p ≤ 0.05 vs. D-gal control; ** *p* ≤ 0.01 vs. D-gal control. D-Gal: D-galactose.

Advanced glycation end products (AGEs) were assessed in serum samples from both non-immunized and immunized groups. The results indicate that the non-immunized D-Gal + UP446 groups had significantly lower serum AGEs compared to the non-immunized D-Gal mice treated with the vehicle. These reductions were dose-correlated, with 33.9% and 58.0% reductions in serum AGE levels for the 100 and 200 mg/kg UP446, respectively (Figure 6). When compared to the normal control, it was found that mice treated with the high dose (200 mg/kg UP446) showed statistically significant reductions.

### 3.8. Effect of UP446 on Antioxidant Enzyme Glutathione Peroxidase In Vivo

**Figure 7:**
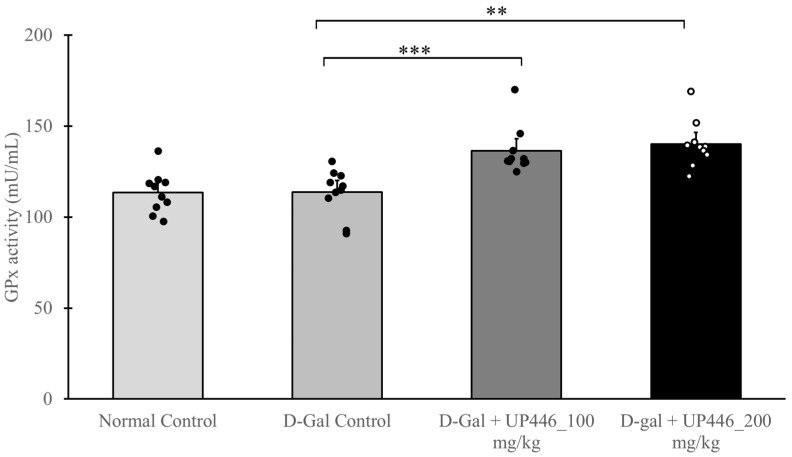
Antioxidation effect of UP446 on immunized mice. Male CD-1 mice (n = 10) received subcutaneous injections of D-galactose at a dose of 500 mg/kg daily for 10 weeks to promote accelerated aging. Starting in week 5, the mice were orally administered two doses of UP446 (100 or 200 mg/kg) and were immunized with 3 μg of the Fluarix quadrivalent vaccine at the end of week 8 (beginning of week 9). A necropsy was performed 2 weeks post immunization, at the conclusion of week 10. The serum was isolated at necropsy and tested for glutathione peroxidase activity using a Glutathione Peroxidase Assay Kit following the manufacturer’s instructions. ** *p* ≤ 0.01 vs. D-Gal control; *** *p* ≤ 0.001 vs. D-Gal control.

Increased levels of antioxidant enzymes suggest that an intervention can improve the host’s ability to counteract excess ROS. The activity of glutathione peroxidase (GSH-Px) was determined in serum samples from immunized mice. Dose-correlated, statistically significant increases of 20.1% and 23.3% in glutathione peroxidase activity were observed in immunized D-Gal mice treated with 100 and 200 mg/kg UP446, respectively (Figure 7). These changes were also statistically significant when they were compared against the normal control for both the dosages.

## 4. Discussion

Immunosenescence, the gradual decline of the immune system associated with aging, is closely linked with oxidative stress. In the context of the respiratory system, oxidative stress exacerbates the decline in immune function by compromising the phagocytosis of immune cells and initiating chronic inflammation. The lungs are especially susceptible to oxidative stress due to constant exposure to environmental pollutants, pathogens, and other harmful agents inhaled during respiration. Immunosenescence further reduces the lungs’ ability to respond to infections and repair damage, increasing vulnerability to respiratory diseases and chronic conditions. Together, oxidative stress and immunosenescence create a vicious cycle, where impaired immune function enhances susceptibility to oxidative damage, and oxidative stress further weakens the immune response. This highlights the importance of interventions to manage oxidative stress and support immune function in aging populations to protect lung health. Herbal medicines are seen as safer alternatives to conventional therapies for respiratory system support; however, most have not been scientifically validated for their safety and effectiveness as alternatives to natural supplements.

The data compiled in this report suggest that UP446 as a natural standardized bioflavonoid composition extracted from roots of *Scutellaria baicalensis* and heartwoods of *Acacia catechu* could potentially disrupt the oxidative stress and immunosenescence perpetual occurrence by mitigating age-associated immune decline and possessing strong antioxidation properties.

UP446 has shown strong antioxidation properties in vitro. The Oxygen Radical Absorbance Capacity (ORAC) measures a substance’s ability to neutralize free radicals. Higher ORAC values suggest a greater capacity to neutralize free radicals. UP446 has been shown to possess high ORAC value at 70,930 (µmole TE) per gram. While the body typically maintains a balance between free radicals and antioxidants, excessive free radical production may require intervention. As such, UP446 could serve as a metabolism “sink” for the excess ROS to minimize their unwanted signaling and tissue damage.

Antioxidants such as UP446 play a crucial role in mitigating the formation and effects of advanced glycation end products (AGEs). By neutralizing reactive oxygen species and inhibiting glycation processes, UP446 helps reduce oxidative stress and AGE accumulation. In this study, age-associated increases in advanced glycation end products (AGEs) were observed in galactose-induced aging mice treated with a vehicle. UP446 significantly decreased the level of AGEs up to 58.0% after oral administration 200 mg/kg.

The accumulation of AGEs accelerates the decline in multiple system functions seen with aging, thus contributing to the aging process [18]. AGEs are known to cause extensive tissue damage by increasing inflammation and oxidative stress. Consistent with our findings, some antioxidants, like resveratrol, have shown potential in preventing AGE formation and breaking existing AGE cross-links, which can alleviate tissue damage and improve vascular health [19]. Therefore, incorporating UP446 into a daily supplement could promote wellness and combat age-related complications linked to AGE accumulation.

Glutathione peroxidase (GPx) is a key endogenous antioxidant enzyme that plays a critical role in protecting cells from oxidative stress [20]. It safeguards the body against oxidative stress-induced cellular dysfunction and contributes to overall health. Its regulation is significant in defending against age-related diseases. D-Gal induced aged mice supplemented with UP446 showed a dose-dependent increase in the level of GPx. Previously, individual components of UP446 have shown a significant increase in the levels of GPx. For example, the antioxidative actions of flavonoids quercetin and catechin were reported through the activation of hepatocyte glutathione peroxidase [21]. Similarly, multiple studies have shown the impact of baicalin in reversing the oxidative stress-induced reduction of glutathione peroxidase [22,23]. The results observed in our study could be the combined and synergistic effect of those two bioactive flavonoids in stimulating the production of GPx.

As maintaining adequate GPx function is crucial for supporting optimal antioxidant defense mechanisms and mitigating oxidative stress-related health risks, the inclusion of UP446 as a dietary supplement could provide significant benefits for overall health.

The NF-kB as a transcription factor complex is one of the cellular sensors that responds to oxidative stress and regulates gene expression [24]. Oxidative stress and the NF-κB signaling pathway are closely interconnected and significantly influence the aging process [25]. Reactive oxygen species (ROS) from oxidative stress activate NF-κB, a transcription factor that drives the gene expressions of many proinflammatory cytokines. This leads to chronic sterile inflammation, a key feature of aging and related chronic diseases. The feedback loop between ROS and NF-κB exacerbates oxidative damage and inflammation, contributing to immune cellular senescence and tissue function decline.

In the current study, aged D-gal mice treated with a vehicle showed a significant increase in the expression of NF-kB. In contrast, when these aged mice were orally administered UP446, the expression of NF-kB was significantly reduced. In agreement with our findings, it was previously reported that the inflammatory gene transcriptional factor controller, NF-κB, was down-regulated by UP446 in LPS-induced cellular models, highlighting the anti-inflammatory activities of UP446 [26]. Therefore, targeting oxidative stress and NF-κB activity through supplementation with UP446, which possesses antioxidant and anti-inflammatory properties, could promote healthy aging and mitigate age-related chronic diseases.

Repeated subcutaneous injections of D-galactose into mice induces significant oxidative stress that causes the damage of immune organ such as thymus, resembling alterations that take place during the normal aging process. A greater thymus index is associated with a more robust immune response [27]. The thymus indices for the normal control group and both UP446 + D-gal treatment groups were significantly greater than those of the vehicle + D-gal group, indicating that these treatments protect this immune organ from senescence induced by oxidative stress, which may result in enhanced production and maturation of immune cells. Supporting our findings with this model, Wei et al. reported that resveratrol promotes the proliferation of immune cells by improving thymus senescence and preventing involution [28].

Supplementation with the bioflavonoid composition UP446 induced mucosal immunity, in particular by increasing the production of IgA while also increasing IgG levels. This indicates the potential use of UP446 for lung protection and the preservation of mucosal immunity. A declined immune response, as reflected by reduced antibody production (IgA and IgG) and suppressed innate (NK cells) and adaptive immune cells (T cells and cytotoxic T cells), has been documented in the Endoxan-induced immune suppression and accelerated aging models. The reduced efficiency of both innate and adaptive immune responses, characterized by weakened macrophage activity, lower cytokine and antibody production, and impaired inflammatory regulation, are among the key features of immunosenescence. Supplementation with UP446 has reversed these age-associated and chemically induced immune senescence.

Specifically, the increase in IgA is crucial for the mucosal protection of the respiratory system, as this immunoglobulin is recognized for its ability to protect mucosal surfaces from microorganisms and foreign antigens. It also neutralizes bacterial products and helps eliminate pathogens or antigens that have penetrated the mucosal barrier [29]. These preclinical findings were later corroborated in a randomized, triple-blind, placebo-controlled clinical trial, where total IgA and IgG levels were found to be increased in senior participants who were orally supplemented with UP446 250 mg b.i.d compared to those on placebo. In the same clinical study, Lewis et al. reported that serum glutathione peroxidase (GSH-Px) was increased as a result of 500 mg/day UP446 supplementation, supporting the current preclinical antioxidation findings [30].

Herbal medicine is commonly used to support respiratory tract health during colds and flu. Among herbal remedies, echinacea, garlic, ginger, and elderberry have been used as complementary approaches to managing respiratory infections, with the potential to enhance immune function, reduce symptom severity, and shorten illness duration [31]. While some herbal treatments show notable promise, the scientific evidence varies, and their effectiveness may differ among individuals. In this regard, *Scutellaria baicalensis* has shown promise for treating the common cold, fever, and influenza. Research on cell cultures and mice has demonstrated that baicalin (the major active component of UP446) exhibits significant antiviral activity, particularly affecting virus budding in a dose-dependent manner. It functions as a neuraminidase inhibitor and is effective against various strains of influenza A virus both in vitro and in vivo [32]. The findings suggest that baicalin could be a valuable treatment option for influenza virus infections. In fact, a recent list of the top 30 herbs in Traditional Chinese Medicine (TCM) for treating respiratory infections ranked Radix *Scutellariae* as the second most utilized herb, with a 38% frequency in all TCM compositions for the treatment of respiratory tract infections [33]. We believe that the current findings depicted in this report will provide additional benefits in expanding the possible mechanisms of action of this botanical application, strengthening its traditional use for respiratory system support.

While the data depicted in this report are promising, this study and its interpretations are not without limitations. The thymus indices have not been confirmed with histopathology, leaving the question of immune organ protection by the supplement unresolved. Additionally, for NFkB analysis, the groups were run separately on SDS-PAGE, which casts doubt on the relative efficacy analysis data. Moreover, despite genetic and physiological similarities, mice and humans differ in immune system functioning and responses to pathogens. Although both species exhibit common aging signs, such as reduced immune function and increased susceptibility to diseases, the specific mechanisms and manifestations of aging can differ. These disparities can affect the accuracy of translating findings from mouse models to human conditions. Additionally, the controlled laboratory environment of mouse studies often does not account for the complex interactions and variability found in human populations, such as genetic diversity, environmental factors, and comorbid conditions. Consequently, while mouse models provide valuable insights, their results need to be carefully validated through human clinical trials before drawing definitive conclusions.

## 5. Conclusions

Collectively, supplementation with the standardized bioflavonoid composition of not less than 60% baicalin from the roots of *Scutellaria baicalensis* and not less than 10% catechin from the heartwoods of *Acacia catechu* has resulted in the protection of immune senescence from oxidative stress. In addition to the previously reported traditional usage, extracellular HMGB1 suppression activity and the increase in IgA from the human clinical trial, the data depicted in this report suggest that supplementation with UP446 could be beneficial for respiratory system support during immune-stressing conditions caused by aging, oxidative stress, and/or pathogen challenges.

## Figures and Tables

**Table 1:** Oxygen radical absorbance capacity of UP446.

Material	Specification	Peroxyl Radicals	Hydroxyl Radicals	Peroxy Nitrite	Super Oxide Anion	Singlet Oxygen	ORAC 5.0 (Total Score)
UP446	≥60% baicalin ≥10% catechins	8375	55,660	1397	4767	731	70,930

**Table 2:** Effect of UP446 on circulating immune cell populations in immunized mice †.

Cell Type	Normal Control	D-Gal Control	UP446_ 100 mg/kg	UP446_ 200 mg/kg
Lymphocytes	98.1 ± 0.9	98.8 ± 0.9	98.4 ± 1.2	98.2 ± 1.6
T cells	13.3 ± 3.1	13.0 ± 2.5	13.4 ± 3.3	15.8 ± 3.4 *
Helper T cells	8.5 ± 1.9	8.3 ± 1.5	8.0 ± 2.5	9.6 ± 2.5
Cytotoxic T cells (% of CD45+ cells)	4.2 ± 1.4	4.0 ± 1.2	4.4 ± 1.4	5.5 ± 1.3 *
Natural killer cells	4.2 ± 0.8	3.4 ± 1.7	4.8 ± 1.1 *	3.7 ± 2.1
Granulocytes	17.9 ± 6.7	23.1 ± 5.1	20.7 ± 4.4	20.0 ± 4.8
B cells	56.3 ± 7.1	50.4 ± 4.4	53.2 ± 6.5	51.2 ± 5.5
Gamma delta T cells	0.35 ± 0.15	0.38 ± 0.14	0.62 ± 0.81	0.33 ± 0.17
CD4+ Gamma delta T cells	0.15 ± 0.08	0.12 ± 0.08	1.00 ± 2.40	0.24 ± 0.20
CD8+ Gamma delta T cells	0.14 ± 0.12	0.12 ± 0.10	0.17 ± 0.18	0.13 ± 0.07

† values are expressed as % of total live cells; * *p* ≤ 0.05.

**Table 3:** Effect of UP446 on circulating immune cell populations in non-immunized mice †.

Cell Type	Normal Control	D-Gal Control	UP446_ 100 mg/kg	UP446_ 200 mg/kg
Lymphocytes	93.5 ± 2.7	97.0 ± 1.9	96.8 ± 1.7	96.1 ± 2.7
T cells	12.9 ± 1.9	10.6 ± 2.6	11.4 ± 2.6	10.1 ± 3.7
Helper T cells	8.9 ± 1.4	6.7 ± 1.8	7.5 ± 1.8	6.4 ± 2.0
Cytotoxic T cells	3.2 ± 1.0	3.2 ± 0.9	3.3 ± 0.9	3.1 ± 1.7
Natural killer cells	4.9 ± 2.0	4.0 ± 1.8	4.9 ± 1.6 *	5.7 ± 1.2 *
Granulocytes	20.0 ± 3.3	23.1 ± 5.2	24.2 ± 11.1	21.6 ± 7.4
B cells	51.0 ± 3.7	51.9 ± 6.3	48.9 ± 10.1	51.7 ± 8.9
Gamma delta T cells	0.65 ± 0.83	0.35 ± 0.14	0.29 ± 0.11	0.33 ± 0.08
CD4+ Gamma delta T cells	2.08 ± 5.98	0.25 ± 0.20	0.13 ± 0.09	0.22 ± 0.14
CD8+ Gamma delta T cells	0.11 ± 0.07	0.13 ± 0.08	0.07 ± 0.09	0.14 ± 0.11

† values are expressed as % of total live cells; * *p* ≤ 0.05.

## Data Availability

Data is contained within the article.

## References

[1] Hou T., Zhu L., Wang Y., Peng L. (2024). Oxidative stress is the pivot for PM2.5-induced lung injury. Food Chem. Toxicol..

[2] Entezari M., Javdan M., Antoine D.J., Morrow D.M., Sitapara R.A., Patel V., Wang M., Sharma L., Gorasiya S., Zur M. (2014). Inhibition of extracellular HMGB1 attenuates hyperoxia-induced inflammatory acute lung injury. Redox Biol..

[3] Patel V., Dial K., Wu J., Gauthier A.G., Wu W., Lin M., Espey M.G., Thomas D.D., Ashby C.R., Mantell L.L. (2020). Dietary Antioxidants Significantly Attenuate Hyperoxia-Induced Acute Inflammatory Lung Injury by Enhancing Macrophage Function via Reducing the Accumulation of Airway HMGB1. Int. J. Mol. Sci..

[4] Wang M., Gauthier A., Daley L., Dial K., Wu J., Woo J., Lin M., Ashby C., Mantell L.L. (2019). The Role of HMGB1, a Nuclear Damage-Associated Molecular Pattern Molecule, in the Pathogenesis of Lung Diseases. Antioxid. Redox Signal..

[5] Yimam M., Horm T., O’Neal A., Jiao P., Hong M., Brownell L., Jia Q., Lin M., Gauthier A., Wu J. (2023). A Standardized Botanical Composition Mitigated Acute Inflammatory Lung Injury and Reduced Mortality through Extracellular HMGB1 Reduction. Molecules.

[6] Hajam Y.A., Rani R., Ganie S.Y., Sheikh T.A., Javaid D., Qadri S.S., Pramodh S., Alsulimani A., Alkhanani M.F., Harakeh S. (2022). Oxidative Stress in Human Pathology and Aging: Molecular Mechanisms and Perspectives. Cells.

[7] Guo J., Huang X., Dou L., Yan M., Shen T., Tang W., Li J. (2022). Aging and aging-related diseases: From molecular mechanisms to interventions and treatments. Signal Transduct. Target. Ther..

[8] Teissier T., Boulanger E., Cox L.S. (2022). Interconnections between Inflammageing and Immunosenescence during Ageing. Cells.

[9] Luo J., Mills K., le Cessie S., Noordam R., van Heemst D. (2020). Ageing, age-related diseases and oxidative stress: What to do next?. Ageing Res. Rev..

[10] Espino J., Pariente J.A., Rodríguez A.B. (2012). Oxidative stress and immunosenescence: Therapeutic effects of melatonin. Oxid. Med. Cell Longev..

[11] Gao Z., Huang K., Yang X., Xu H. (1999). Free radical scavenging and antioxidant activities of flavonoids extracted from the radix of *Scutellaria baicalensis* Georgi. Biochim. Biophys. Acta (BBA) Gen. Subj..

[12] Paudel K.R., Kim D.W. (2020). Microparticles-Mediated Vascular Inflammation and its Amelioration by Antioxidant Activity of Baicalin. Antioxidants.

[13] Bai X., Yao M., Zhu X., Lian Y., Zhang M. (2023). Baicalin suppresses interleukin-1β-induced apoptosis, inflammatory response, oxidative stress, and extracellular matrix degradation in human nucleus pulposus cells. Immunopharmacol. Immunotoxicol..

[14] Bernatoniene J., Kopustinskiene D.M. (2018). The Role of Catechins in Cellular Responses to Oxidative Stress. Molecules.

[15] Jia Q. (2009). Formulation of a Mixture of Free-B-Ring Flavonoids and Flavans as a Therapeutic Agent. U.S. Patent.

[16] Yimam M., Zhao Y., Ma W., Jia Q., Do S.-G., Shin J.-H. (2010). 90-day oral toxicity study of UP446, a combination of defined extracts of *Scutellaria baicalensis* and *Acacia catechu*, in rats. Food Chem. Toxicol..

[17] Feng H., Fan J., Lin L., Liu Y., Chai D., Yang J. (2019). Immunomodulatory Effects of Phosphorylated *Radix Cyathulae officinalis* Polysaccharides in Immunosuppressed Mice. Molecules.

[18] Semba R.D., Nicklett E.J., Ferrucci L. (2010). Does accumulation of advanced glycation end products contribute to the aging phenotype?. J. Gerontol. A Biol. Sci. Med. Sci..

[19] Maleki V., Foroumandi E., Hajizadeh-Sharafabad F., Kheirouri S., Alizadeh M. (2022). The effect of resveratrol on advanced glycation end products in diabetes mellitus: A systematic review. Arch. Physiol. Biochem..

[20] Averill-Bates D.A. (2023). The antioxidant glutathione. Vitam. Horm..

[21] Nagata H., Takekoshi S., Takagi T., Honma T., Watanabe K. (1999). Antioxidative action of flavonoids, quercetin and catechin, mediated by the activation of glutathione peroxidase. Tokai J. Exp. Clin. Med..

[22] Ma L., Wu F., Shao Q., Chen G., Xu L., Lu F. (2021). Baicalin Alleviates Oxidative Stress and Inflammation in Diabetic Nephropathy via Nrf2 and MAPK Signaling Pathway. Drug Des. Dev. Ther..

[23] Fang J., Wang H., Zhou J., Dai W., Zhu Y., Zhou Y., Wang X., Zhou M. (2018). Baicalin provides neuroprotection in traumatic brain injury mice model through Akt/Nrf2 pathway. Drug Des. Dev. Ther..

[24] Helenius M., Hänninen M., Lehtinen S.K., Salminen A. (1996). Aging-induced up-regulation of nuclear binding activities of oxidative stress responsive NF-kB transcription factor in mouse cardiac muscle. J. Mol. Cell. Cardiol..

[25] Galli F., Marcantonini G., Giustarini D., Albertini M.C., Migni A., Zatini L., Gioiello A., Rossi R., Bartolini D. (2022). How Aging and Oxidative Stress Influence the Cytopathic and Inflammatory Effects of SARS-CoV-2 Infection: The Role of Cellular Glutathione and Cysteine Metabolism. Antioxidants.

[26] Tseng-Crank J., Sung S., Jia Q., Zhao Y., Burnett B., Park D.R., Woo S.S. (2010). A medicinal plant extract of Scutellaria Baicalensis and *Acacia catechu* reduced LPS-stimulated gene expression in immune cells: A comprehensive genomic study using QPCR, ELISA, and microarray. J. Diet. Suppl..

[27] Thapa P., Farber D.L. (2019). The Role of the Thymus in the Immune Response. Thorac. Surg. Clin..

[28] Wei T.T., Li M.J., Guo L., Xie Y.D., Chen W.H., Sun Y., Liu G.H., Ding Y., Chai Y.R. (2020). Resveratrol ameliorates thymus senescence changes in D-galactose induced mice. Microbiol. Immunol..

[29] Pilette C., Ouadrhiri Y., Godding V., Vaerman J.P., Sibille Y. (2001). Lung mucosal immunity: Immunoglobulin-A revisited. Eur. Respir. J..

[30] Lewis E.D., Crowley D.C., Guthrie N., Evans M. (2023). Role of *Acacia catechu* and *Scutellaria baicalensis* in Enhancing Immune Function Following Influenza Vaccination of Healthy Adults: A Randomized, Triple-Blind, Placebo-Controlled Clinical Trial. J. Am. Nutr. Assoc..

[31] Mousa H.A. (2017). Prevention and Treatment of Influenza, Influenza-Like Illness, and Common Cold by Herbal, Complementary, and Natural Therapies. J. Evid. Based Complement. Altern. Med..

[32] Ding Y., Dou J., Teng Z., Yu J., Wang T., Lu N., Wang H., Zhou C. (2014). Antiviral activity of baicalin against influenza A (H1N1/H3N2) virus in cell culture and in mice and its inhibition of neuraminidase. Arch. Virol..

[33] Ge H., Wang Y.F., Xu J., Gu Q., Liu H.B., Xiao P.G., Zhou J., Liu Y., Yang Z., Su H. (2010). Anti-influenza agents from Traditional Chinese Medicine. Nat. Prod. Rep..

